# Peer-Mediated Theatrical Engagement for Improving Reciprocal Social Interaction in Autism Spectrum Disorder

**DOI:** 10.3389/fped.2014.00110

**Published:** 2014-10-10

**Authors:** Blythe A. Corbett, Lydia R. Qualls, Blythe Valencia, Stéphanie-M. Fecteau, Deanna M. Swain

**Affiliations:** ^1^Department of Psychiatry, Vanderbilt University, Nashville, TN, USA; ^2^Vanderbilt Kennedy Center, Nashville, TN, USA; ^3^Vanderbilt Child Studies, Nashville, TN, USA; ^4^Virginia Polytechnic Institute and State University, Blacksburg, VA, USA

**Keywords:** autism, peer mediation, social skills, active learning, theatre, context

## Abstract

The hallmark characteristic of autism spectrum disorder (ASD) is poor reciprocal social communication. Interventions designed to improve this core deficit are critically needed. Social skills interventions such as direct training, peer mediation, and video modeling have contributed to improvements in various social skills in children with ASD. This paper reviews existing social competence interventions available for children with ASD while highlighting hypothesized critical components for advancing, maintaining, and generalizing skills, which include (1) peer mediation, (2) active learning, and (3) implementation in supportive, natural contexts. As a framework for these approaches, this conceptual paper describes SENSE Theatre, a novel intervention that combines trained peers that facilitate the performance-based theatrical treatment delivered in a supportive, community-based environment. A review of previous research shows early feasibility, setting the stage for more rigorous studies to aid in developing a standardized intervention package.

## Introduction

Children with autism spectrum disorder (ASD) have difficulty with social interaction, communication, and responding in a flexible way to everyday life (1). A recent report suggested that 1 in 68 individuals are now affected by this complex neurodevelopmental disorder (2). Affected individuals display a wide range of ability in language, sociability, and intellectual functioning. However, the defining characteristic across all levels of functioning is impairment in reciprocal social interaction, which can manifest as limitations in many areas of functioning including social-emotional reciprocity, to-and-fro conversation, play behavior, use of non-verbal communication, and in the development of age-appropriate relationships (1). There is significant diversity in symptom presentation, which can range from spontaneous social approaches with poor understanding of social rules to avoidant behavior with little evidence of social interest (3–5). Similarly, there is significant diversity in repetitive and stereotyped patterns of behavior (6) and a range of hyper- and hypo-sensitivity to sensory experiences (7).

Interacting with others relies on the coordinated effort of many complex and integrated skills to include the ability to recognize, identify, integrate, plan, and respond appropriately to dynamic socioemotional information. This information is conveyed, in part, through the interpretation and expression of verbal (e.g., speech prosody) and non-verbal (e.g., facial expressions and gestures) forms of communication (8), which can be difficult for those affected by ASD to interpret. Additionally, many individuals with ASD have difficulty perceiving, understanding, and remembering emotions (9, 10) and human faces (11–17). In addition to understanding how others feel, children with ASD have limitations in empathizing and predicting how others will think and act (18). Understanding other people’s thoughts and feelings is a part of the concept of “theory of mind,” which is defined as the ability to attribute mental states, such as beliefs, to other people and then use these ideas to understand and predict other peoples’ behavior (18–20). Since sociability is a fundamental and encompassing skill, interventions designed to improve the complexity and diversity of social functioning are critically needed.

During social interaction with peers, many children with ASD experience heightened physiological stress as measured by cortisol, a primary stress hormone in human beings (21–24). Moreover, many children and adolescents with ASD show significant anxiety in social contexts especially as they get older and gain insight into their challenges engaging with others (25, 26). Despite showing improvements in social communication through experience and development, many children with ASD display higher levels of stress as measured by increased cortisol during benign social interactions with same age peers (22, 23, 27). It has been shown that behavioral patterns of approach and avoidance, as well as age factors can contribute to distinct patterns of stress in youth with ASD (22). Thus, stress responsivity may be an important moderator of social interaction for children with ASD and should be considered in programs developed to improve social skills (28, 29).

Currently, there are no known pharmaceutical treatments that have been shown to improve the core symptoms of ASD; thus, behavioral-based interventions designed to improve social skills are essential. There are many different types of social skills interventions available including direct skills training, applied behavior analysis, social skills groups, video modeling, and peer training (30–35). Many training programs produce positive outcomes in children with ASD; however, only a few meet strict criteria for empirical support (e.g., inclusion of control groups and randomization) (33) in order to distinguish observed effects attributed to the intervention from natural improvements over time (36–38).

## Background

The current paper briefly introduces some existing social skills interventions available for children with ASD that are relevant to the concepts outlined below. Comprehensive reviews of social skills program and treatment options for children with ASD are more thoroughly reviewed elsewhere [e.g., Ref. (38–41)]. Teaching appropriate social skills to children with ASD by direct didactic training in either a group or one-on-one format has received empirical support (33, 42, 43). Qualified psychologists and paraprofessionals often teach these training sessions in clinic settings, which have demonstrated immediate, in-context gains for targeted skills. While participating children with ASD may learn specific social skills such as initiating interaction using priming (35), improving conversation skills through direct instruction with caregivers (44), or enhancing perspective taking from teaching theory of mind and social skills (45), acquired skills often do not generalize across people, contexts, or everyday environments (38, 46). Thus, the strong context-dependent learning in children with ASD is the most common barrier to behavioral change in children and adolescents with ASD (47). Thus, the inclusion of typically developing peers in the training and active practice of social skills can significantly contribute to the acquisition, maintenance, and generalization of sociability. Moreover, as described below, the context in which treatment is delivered also can impact social skill development.

Rather than reviewing or critiquing a broad range of social skills programs, the strengths of the approaches that are relevant to our theatre-based social engagement treatment are highlighted. In the context of the paper, three key components are discussed that we hypothesize to be necessary for advancing, maintaining, and generalizing social interaction abilities in children and youth with ASD, which include (1) peer mediation, (2) active learning, and (3) implementation in supportive, natural contexts. Moreover, these central ideas involve the inclusion of the recipient or target of the treatment in the intervention model to achieve changes in self-efficacy. Additionally, the conceptual paper presents SENSE Theatre, a novel intervention that combines trained peers that facilitate the performance-based theatrical approach delivered in a supportive, community-based environment.

## Peer Mediation

Peers can have a profound impact on the psychological, social, and physiological functioning of other children, including children with ASD (22–24, 27). Peer-mediated interventions utilize typically developing peers as agents of change, facilitating the development of social behavior and engagement in children with ASD (48–50). Peer mediation programs vary in the degree to which peers are trained in the delivery of social skills. One widely used peer-mediated program for children with ASD in school settings involves a one-on-one assistant who helps the child throughout the school day and is given the task of helping the child integrate socially with others (51). Although well intended, if the peers are untrained, the approach may produce unwelcome effects as it may label the child with social deficits as “different,” possibly leading to stigmatization and greater social isolation. Peers’ negative expectations of the target children must be managed in order to prevent this effect, and peer training is an effective way to achieve more positive expectations (52). For example, studies utilizing students who participated in trained mediation groups show greater generalization of skills for children with ASD (53). Peers should not simply be a social partner but also an active model of appropriate social behavior (54). In some programs, peers are extensively trained in how to elicit certain target behaviors through modeling, reinforcement, scripts, and other program-specific methods (55, 56). Thus, peers may serve as the optimal agent of change because they are not only the interventionists but they are also the intended recipients of improved socialization.

Since the primary objective for any social skills program is for children to be able to interact more competently with peers in natural settings (52), the inclusion of trained peers in treatment is both logical and beneficial. Moreover, it may be economical; utilizing peers as a resource for learning has the potential for reducing the demands on teachers and parents (57). Utilizing peers increases the number of training models for practice (49), which supplements instruction provided by parents and professionals. Zhang and Wheeler (58) performed a meta-analysis of peer-mediated interventions for children aged 8 years and younger and found them highly effective for increasing social interactive behavior. Additionally, Lang et al. (57) performed an analysis of several studies that utilized recess time to improve social skills and found that use of peer mediation increased social interaction for children with ASD. Further, peers participating as mediators can increase peer acceptance and engagement with others, and this in turn can be a powerful agent of change for children with ASD (51).

Peer-mediated interventions not only benefit the child with ASD but they also provide positive results for the typically developing children delivering the intervention. While it can be argued that school-based peer mediation takes away valuable learning opportunities for those administering the intervention, Hunt and Goetz (59) report that these peers demonstrate positive social-emotional growth and participation does not interfere with academic performance. As an added benefit, participating peers often develop positive and accepting attitudes when they observe the capability of their peers with a disability (60).

Kasari and colleagues (51) performed one of the first randomized controlled studies that compared the effectiveness of one-on-one direct social skills training to a peer-mediated model for high-functioning children with ASD. Peers received extensive instruction (40 min per week for 6 weeks) and training in how to recognize and lend social support to those who may have social difficulties. Results showed that children who received the peer-mediated intervention had increased involvement in classroom social networks as rated by teachers and untrained peers in the classroom, as well as less isolation on the playground. These treatment gains persisted to a 3-month follow-up. Despite these improvements, reciprocal friendships remained low for children with ASD and they still exhibited isolated play on the playground, although to a lesser extent.

While the previous study shows that peer mediation may be an independently superior treatment than simply direct skills training, using the treatments in combination should also be considered when creating an efficacious social skills intervention. Banda et al. (61) conducted a study that combined direct instruction and peer training for increasing social skills in two elementary students with ASD. They had teachers in a classroom setting model to participants and peers how to initiate questions and answer with appropriate responses, while praising students for properly asking and responding to questions. Results showed an increase in participant initiating and responding. A similar study had an interventionist teach a participant and two peers various phrases and skills to promote cooperative play at recess and provide cues during appropriate times of play. All three participants had higher rates of communicative acts during the intervention sessions than at baseline (62). Schmidt and Stichter (63) had adolescent peer mediators interact with three target students with ASD who had just completed a direct skills training program delivered in a school setting and found that the addition of the peer mediators lead to improved skill generalization for all three students with ASD. As these studies illustrate, adult supervision using direct skills training in concert with peer intervention may be a strategic combination for improving the social engagement of children with ASD.

## Social Learning is Active *“Practice is the Key to Social Success”*

Whether by direct skills didactic training or peer-mediated interventions, social learning theory posits that much of human behavior is learned by watching and imitating others (64). In fact, four processes are deemed necessary to promote the acquisition of social behavior, namely, attention, retention, production, and motivation. In other words, despite the misleading title, observational learning is not a passive task of merely watching others; it is an active and engaging process. Thus, programs that include techniques to activate learning and engage the participant through the actual performance and practice of social skills can advance learning (65–68). As discussed below, acting is fundamentally active and thereby may provide the opportunity to enhance observational learning and the practice of social skills.

Reciprocal social interaction requires active engagement and practice. While it is helpful to teach social rules and strategies, there are a variety of possible encounters and, therefore, the proficiency, spontaneity, and improvement in socialization is gained through interacting with a variety of partners. In infancy and early childhood, many fundamental social skills are acquired by imitating others; yet, children with autism have difficulty with imitation (69), which includes knowing who to model, what behaviors to imitate, and in what context to apply the behaviors learned (70). Since imitation is one of the ways in which children learn social rules, early deficits in this ability could impair a child’s social development (71).

As a key component in social learning (64), imitation is not a passive process, rather, it is a volitional, effortful, and active process (72). Studies show that children with autism are impaired in imitating various actions and emotions compared to peers with other developmental delays or typical development (69). In this study, Rogers and colleagues postulate that imitation deficits in children with autism may lead to a lack of active imitation practice due in part to a diminished interest in socially motivating rewards (69). This suggests that in order to engage children with autism to practice imitating others, they must be motivated to observe and imitate the actions of others. One approach shown to capture the motivation of children with ASD is video modeling (31, 73, 74).

Video modeling is an effective way to work with children on the autism spectrum to capture and transfer the targeted skills into their repertoire. In video modeling, an individual watches specific skills presented on a video, then imitates through active practice of the performed skills often in the form of a role-play that may be initially guided by a trained paraprofessional or parent. Video modeling has consistently gained empirical support across a number of studies for improving various aspects of social functioning and teaching adaptive behaviors, such as reciprocal play and promoting social language verbalizations in children with autism (30, 31, 75–81). The use of video can facilitate observational learning and generalization of behavior because it is an inherently motivating medium (28, 31, 73, 74). Since video modeling involves not only watching the performance of others but also includes role-play with another person, it is complimentary to several acting approaches used in theatre outlined below.

## Natural and Supportive Contexts

It is well established that the context in which an individual learns can significantly impact the retention and transfer of skills in typically developing children (82, 83) and ASD (37, 57). Motivation is a key component in active learning, and is influenced by external factors, including the learning context (84). An environment that is perceived as naturally stimulating and supportive leads to greater motivation and participation compared to restrictive and controlled settings (85). This is shown in children with ASD as well as other developmental disabilities demonstrated by Gresham and colleagues (86), who state that social skills interventions with poor generalization outcomes usually take place in more restrictive settings that are unlike the child’s usual environment. In contrast, the positive impact of social skills training with or without peers can be enhanced when conducted in natural settings, thereby leading to greater generalization and maintenance (46, 87, 88). Providing a least restrictive environment allows children of all achievement and developmental needs to receive an adequate and effective education (89). Peer-assisted learning strategies implemented within an inclusive classroom environment can provide reciprocal learning when delivered in a supportive and structured manner (90). In the process, children can benefit from different instructional procedures that are conducive to individual learning needs while allowing the teacher the opportunity to supervise a variety of instructional methods simultaneously (90).

The context in which typically developing peers and children with developmental needs are brought together is especially critical. For example, an increase in communication and interaction was demonstrated in children with ASD utilizing peer mediation at recess where it is possible to *encourage* appropriate social interactions with peers, although the authors acknowledge that such an approach lacks structure and close adult supervision (62). Kasari and Smith (91) propose that contexts outside of school that mimic the school environment could be an effective compromise between the naturalistic environment of a cafeteria or playground and the structure of a clinic setting. Ideally, a combined supportive structure may be the best. For example, Sansosti (92) proposes a multitier framework to teach social skills for children with ASD that includes core (e.g., positive behavior support), targeted (social skills groups), and intensive (individualized videos) intervention strategies.

In treatment, the contextual framework plays an integral role in the development and transfer of skills (37, 57, 82). In order for treatment to be successful, interventionists have the responsibility of cultivating applicable learning environments that will lead to successful dissemination within the community (93). Effective learning conditions ought to be natural, motivating, and supportive (46, 84, 85, 87, 88). Moreover, the study of an intervention needs to be applied within the same context and under the same conditions for which it was developed (93). In short, context is not an extraneous variable, it is a critical factor. The SENSE Theatre intervention discussed below fosters these principal conditions while providing a supportive context in which there is clear structure and one-to-one peer supervision, with many adults in close contact with the participants.

## SENSE Theatre, a Novel, Peer-Mediated, Performance-Based Program Delivered in a Community-Based Intervention

While there is an inherent drive in science, and intervention research in particular, to reveal the key contributing components or mechanisms that lead to change, the treatment of such complex skills will naturally require several components. Nevertheless, we argue that a few important components are critical for the acquisition, maintenance, and generalization of sociability in ASD. As previously described, peer mediation, active practice, and training conducted in a supportive, natural context can significantly impact the acquisition of important reciprocal social interaction skills. Here, we highlight the importance of these techniques that are integral to a novel program called SENSE Theatre that includes these important elements as well as a unique form of treatment delivery, the use of theatre, and acting techniques.

Acting is an interactive process that involves many aspects of socializing, namely, observing, perceiving, interpreting, and expressing thoughts and ideas. For example, an actor must pay attention to the other actors, listen and react to their cues, and express the thoughts and feelings of their characters. It also encompasses other important elements known to be problematic in individuals with ASD, theory of mind and empathy (18, 19). An actor must learn to take on the perspective of another character, which includes their beliefs and feelings. The process can lead to enhanced awareness and increased understanding of the experiences of other people. Thus, in the process, children with ASD may gain greater insight and ability to attribute mental states and feelings onto oneself and others. In fact the inclusion of various theatrical approaches into earlier intervention programs for individuals with typical development and ASD have shown improvement in relevant areas of social functioning, such as empathy and perspective taking (28, 94–98). In the SENSE Theatre program, a variety of acting techniques are employed to include role-play, scripts, and improvisation that provide an opportunity for the participant with ASD to explore and practice social interaction skills in a safe and supportive environment (28, 29). Participants engage in peer-mediated theatre games, such as mirroring, which involve imitating a partner’s actions, thoughts, or feelings. Acting provides the opportunity for the children with ASD to interact with peers and indirectly practice social skills. For example, role-play exercises require verbal and non-verbal turn taking, which can facilitate reciprocal communication and interaction skills. Improvisation is also an active and dynamic process in which scenarios are presented to perform without preparation, which engages the child’s imagination and allows more flexible thought and behavior.

### Peers as an expert model

The SENSE Theatre intervention utilizes highly interactive peers to facilitate social interaction in children with ASD. While there are different types of models that can be employed in treatment studies, the utilization of an expert model can provide direct instruction from persons that characterize optimal functioning. For example, integrated playgroup models have been used in which *expert players* help instruct the *novice player*s on particular skills (99, 100). In the current theatre intervention, each participant with ASD is paired with a typically developing child with acting skills who, in addition to being a co-actor in a play, serves as the participant’s peer model. In the SENSE approach, youth actors are conceptualized as “experts” of reciprocal social interaction skills that include verbal and non-verbal communication, socioemotional perception, and expression, as well as behavioral and affective control. It has been shown that learning from an expert with instructional cues can enhance learning (101). Learning by watching and interacting with the expert models in a to-and-fro social exchange develops the skills in an efficient and precise manner.

In fact, the peers are selected, in part, based on their exceptional skills in key areas of social functioning, which are often found in actors (e.g., empathy, social communication) (102). While testing of peers for inclusion is not required, prior to training, we recently identified some of the underlying characteristics of many of the peers. Based on self-report measures, the majority of peers score highly on empathy (103) and self-efficacy (104). It is likely that these key attributes are fundamental to peer mediation such that strong empathy skills allow the peers to be concerned about the welfare of the child with ASD and that high confidence allows them to serve as an expert model. Although this is currently conjecture, efforts are underway to examine these hypotheses.

Prior to the start of the intervention, peer actors receive comprehensive training in key topics including characteristics of ASD, a variety of established behavioral intervention techniques, and the SENSE Theatre manualized approach. When peer training is included in an intervention, it is important to examine the integrity of the training by testing the acquisition of knowledge [e.g., Ref. (63)]. In the SENSE Theatre program, this is conducted immediately before and after peer training.

Moreover, to ensure that training is implemented appropriately especially when working with peers or students, delivery fidelity is recommended (105, 106). To assess fidelity of implementation of the behavioral techniques and core objectives of the program, raters behaviorally code the peers during semi-structured activities at three time points in the course of the program. For SENSE Theatre, to achieve fidelity, the peer must obtain a minimum performance of skill implantation (e.g., 80%) on each of the techniques and objectives observed. Research personnel supervise the application of the technique using objective and reliable coding throughout the program. If individual peers have difficulty implementing the techniques or maintaining a high level of skill, then booster sessions and individualized feedback are employed.

### Performance

Through *in vivo* and video modeling with expert peer models, children with ASD are given the opportunity to practice and perform the skills necessary for acquisition. One of the goals of repeated performance of newly learned skills is to reach the status of automaticity (107) in which domain-specific skills became a natural part of the child’s repertoire. Moreover, as part of the SENSE Theatre program, the children perform in a multi-context manner, specifically, in the theatre with their peers and practice at home via video modeling (29), and also perform their skills and roles for the public. Thus, the program is performance-based, enhancing the ability of children to exercise their new and developing social skills for each other, as well as for the public in the form of a play.

### Context

The foundational work of Vygotsky has shown that the context in which learning occurs is critical (83). As part of his theory of the zone of proximal development, a child benefits from training that bridges their actual developmental level with his or her potential developmental level. In SENSE Theatre, one of the core principals is to create an environment that provides the opportunity to advance the child’s learning while supporting the child’s current ability. As noted above, motivation is an important part of active learning, which is influenced by the learning context (84). Social interaction with peers can be challenging and stressful for children with ASD; however, they can be motivated by the inclusion of peers, modeling of positive social engagement, and playful group activities. SENSE Theatre, with the inclusion of supportive theatre games and peers, is naturally stimulating thereby contributing to enhanced motivation and participation (85).

The SENSE Theatre program has been conducted in natural, community-based environments that are supportive and less restrictive, which likely contributes to greater maintenance and generalization of learned skills (46, 87–90). Training is implemented in a hierarchical manner via supervised large group, small groups, and one-on-one peer-mediated teaching allowing individualized support, as needed (92). Additionally, snack breaks are provided fostering less formal engagement and *in vivo* modeling. Thus, the delivery of treatment in a context outside of the child’s school; yet, in the community or school theatre merges the feel of a natural setting with the oversight of a clinical setting (91). Video modeling approaches allow participants to view and practice daily with videotaped peers from home via a password protected website to extend the learning practice (29). Finally, the program includes multiple trained peers that engage directly or indirectly with the participants; as such, the variability and diversity of modeled behavior is more representative of natural social interactions (108).

Thus, acting and the theatrical techniques used in the SENSE Theatre program help to teach social skills through the use of expert peers models who provide multiple opportunities for socialization in real and imagined supportive contexts. Children with ASD require clear and repetitive exemplars, which are delivered in the role-plays and exercises. Simultaneously, the use of improvisation and theatre games stimulates imagination and fosters more flexible thinking and behavior. Moreover, the positive reinforcement received from the peers can enhance social motivation to engage with others. Since ASD has been conceptualized as a disorder of social motivation (3, 4), treatments that show the benefits of socialization set the stage for targeting a core deficit. Acting is interactive, a dynamic process that can fundamentally enhance the attention to, practice of, and motivation to engage in reciprocal social interaction.

## Research Findings

The SENSE Theatre intervention has been offered to dozens of children with ASD and formally examined in two published studies that included a pre-test, post-test design in which measures were assessed immediately before and following the intervention (28, 29). Briefly, the first study was conducted in a community theatre model including approximately 40 intervention hours delivered over a 2 1/2-month duration. Practices were held approximately one to two nights per week including several days of rehearsal the week before the public performance. Participants included eight children with ASD (seven boys and one girl) ranging in age from 7 to 18 years that were paired with typically developing peers. Dependent measures included neuropsychological, biological (cortisol and oxytocin), and behavioral scales to assess social perception, hormone levels, and adaptive behaviors. Primary dependent neuropsychological measures included subtests from the NEPSY (109) to measure memory for faces, affect recognition, and theory of mind, all hypothesized to show skill improvement following the intervention. Cortisol, a measure of stress responsivity, was hypothesized to show initial rise followed by gradual decline as the participants acclimated to the social intervention. The participants with ASD showed moderate improvement in face identification and theory of mind skills following the intervention. Additionally, they demonstrated a reduction in cortisol levels over time (28). The findings suggested changes in social perception and adaptation to the social environment.

Recently, we reported findings on the implementation of a summer camp model that included a comparable dose of 10 sessions. However, it was conducted over a shorter duration and more concentrated dose intensity. Specifically, the treatment was delivered over 2 weeks for 3 1/2 h per day in a summer camp model. Sixteen youth with ASD between 8 and 17 years completed the treatment and 12 participated in the research. A multilevel approach was again employed in which examination of neuropsychological, biological, and behavioral variables was measured. Significant differences in social perception on the NEPSY (109) (memory for faces), social function on the Social Responsiveness Scale (SRS), (110) (awareness, cognition), and adaptive skills on the Adaptive Behavior Assessment System (ABAS (111)) (home living, self-care) were observed. Stress reduction as measured by salivary cortisol was also observed (29).

Since this initial model was implemented, subsequent camp interventions have been delivered using a pre-test, post-test design. In a combined cohort of participants (*N* = 20) from the 2012 and 2013 SENSE Theatre summer camps, several findings were replicated using paired samples *t*-tests. Specifically, social perception in the form of memory for faces immediate [*t*(18) = −2.612, *p* = 0.018] and memory for faces delayed [*t*(18) = −4.194, *p* = 0.001] showed significant change upon post testing. In regard to generalized social functioning based on parental report using the SRS (110), improvement was reported on total social responsiveness [*t*(19) = 2.665, *p* = 0.015], and social cognition [*t*(19) = 3.523, *p* = 0.002] in particular. These results further support the findings that the SENSE Theatre intervention produces improvement in core areas of functioning for many children with ASD, namely, gains in social cognition, memory, and behavior. Moreover, in the most recent data, increases in adaptive skills were reported for functional academics [*t*(18) = −2.617, *p* = 0.017] and self-direction [*t*(18) = −2.179, *p* = 0.043] suggesting that the treatment has positive impact beyond the targeted social communication skills.

Improvements in social functioning have likewise been reported in other peer-mediated social skills interventions previously mentioned (51, 57, 58). In addition, the randomized social skills program given by Solomon and colleagues (33), which targeted emotional awareness, theory of mind, and problem solving, led to improvement in social perception. Also, another program that used theatre techniques to improve social skills (96) similarly found increases in social perception, social assertiveness, and reduction in social problems.

The observed gains in children with ASD in social perception, social functioning, and adaptive skills, which are important building blocks of reciprocal social interaction, provide support for the utilization of theatre-based approaches. While randomized experimental studies are warranted, these preliminary studies collectively provide strong support for the SENSE Theatre model (28, 29).

## Discussion

The hallmark characteristic of ASD is poor reciprocal social communication and a variety of interventions have been employed to improve functioning in these critical skills. Promising approaches, such as peer mediation have contributed to improvements in various specific social skills in children with ASD. The inclusion of typically developing peers in social interaction programs can improve social development and engagement in children with ASD (48–50). Peers can be trained to elicit target behaviors using behavioral approaches such as modeling, reinforcement, and scripting that can enhance generalization (53, 55, 56). In addition to working *in vivo*, video modeling has been shown to be a promising approach to teach a variety of skills to children with ASD (30, 31, 75, 76, 80, 112, 113).

The aforementioned efficacious behavioral approaches have been combined with performance-based theatrical techniques in SENSE Theatre to address barriers to behavioral change and explicitly advance the learning and application of social functioning in youth with ASD. While theatrical techniques such as role playing, improvisation, and character development are rarely used in the treatment of ASD, they have been shown to target core deficits in reciprocal social communication (28, 29, 96, 114). Acting is explicitly active, requiring the performance of thoughts, ideas, and actions thereby providing the child with ASD the opportunity to practice social skills in a supportive albeit dynamic setting. As in acting, interacting with others relies on the ability to recognize, identify, integrate, plan, and respond appropriately to dynamic socioemotional information; therefore, utilizing theatre techniques provides the opportunity to learn and practice these social skills. The trained typically developing peer actors are the primary agents of change and in the process serve as both teacher and recipient of reciprocal social exchange. The program is also enhanced by treatment delivery in a community setting, demonstrating that promising interventions can be delivered in natural contexts while maintaining the rigor of a clinic setting.

The SENSE Theatre model suggests that the inclusion of these approaches in combination with the core theatre methods enhances the training, social experience, and motivation of the participants to engage with peers (28, 29). Combining treatments that have been independently effective has been shown to demonstrate even greater gains when delivered in combination (115). While previous efforts lend preliminary support for SENSE Theatre for improving social interaction skills in ASD, we are conducting a randomized, waitlist control group study with a large sample of participants enrolled in a 10-week model of the SENSE Theatre treatment. Additionally, a manual is in final development, which will allow the treatment to be delivered systematically by other interventionists in various settings to increase reliability and determine efficacy. Some may insist on trying to discover the turnkey ingredient that contributes to the promising results for this or any other treatment. For a complex set of behaviors, such as reciprocal social skills, it is highly unlikely that a single basic factor will be identified to target or explain the benefits when remediated. Indeed, the aforementioned theatrical treatment framework provides evidence that synthesizing peer mediation, active learning, and a supportive, natural context can promote the advancement, maintenance, and generalization of skills for youth with ASD.

## Concluding Remarks

Interventions aimed at improving core impairment in reciprocal social communication in children with ASD are critically needed. Evidence is presented highlighting important components for advancing, maintaining, and generalizing social competence, which include peer mediation, active learning, and the implementation of interventions in supportive, natural contexts. Moreover, acting specifically targets the core ASD impairment in reciprocal social communication and rigid, inflexible thought and behavior by having the child actively engage in activities such as role-playing and improvisation to develop these skills. SENSE Theatre, a promising treatment for youth with ASD, serves as a model for utilizing these elements by combining trained peers that facilitate the performance-based theatrical treatment delivered in a supportive, community-based environment.

## Author Contributions

Blythe A. Corbett conceptualized the paper, developed the SENSE Theatre program, wrote the initial draft of the paper, and revised the final manuscript. Lydia R. Qualls researched peer mediation and active practice, assisted with writing the first full draft, and revised and copyedited the final version of the manuscript. Blythe Valencia researched the importance of context and natural environments in learning, as well as the section on peers as expert models, and helped draft those sections of the manuscript. Stéphanie-M. Fecteau contributed background information regarding autism spectrum disorders and peer mediation, and helped with the revision of the manuscript. Deanna M. Swain contributed to early ideas regarding peer mediation and video modeling strategies for ASD and contributed to the original draft of the paper. All authors have read the final work and agree to be accountable for its accuracy and integrity.

## Conflict of Interest Statement

The authors declare that the research was conducted in the absence of any commercial or financial relationships that could be construed as a potential conflict of interest.
